# Prevalence and prognostic significance of main metabolic risk factors in primary biliary cholangitis: a retrospective cohort study of 789 patients

**DOI:** 10.3389/fendo.2023.1142177

**Published:** 2023-11-08

**Authors:** Dan-Tong Zhao, Hui-Ping Yan, Ying Han, Wei-Ming Zhang, Yan Zhao, Hui-Yu Liao

**Affiliations:** ^1^ Clinical Laboratory Center and Clinical Research Center for Autoimmune Liver Disease, Beijing You’An Hospital, Capital Medical University, Beijing, China; ^2^ Second Department of Liver Disease Center, Beijing You’An Hospital, Capital Medical University, Beijing, China; ^3^ Department of Clinical Laboratory Diagnosis, Beijing You’An Hospital, Capital Medical University, Beijing, China; ^4^ Clinical Laboratory Center, Beijing Chest Hospital, Capital Medical University, Beijing, China

**Keywords:** primary biliary cholangitis, metabolic syndrome, hyperlipidemia, hypertension, type 2 diabetes mellitus

## Abstract

**Background:**

Metabolic risk factors in primary biliary cholangitis (PBC) have not been well described in China. Additionally, it is unclear whether these factors have an impact on the prognosis of PBC patients. Therefore, this study aimed to investigate the prevalence of main metabolic risk factors in PBC, and to evaluate their prognostic values for liver-related outcomes.

**Methods:**

A cohort of 789 PBC patients was retrospectively studied between July 2008 and September 2019 by investigating the main metabolic risk factors and analyzing liver-related outcomes.

**Results:**

At presentation, 271 (34.3%) patients had concomitant hyperlipidemia, 126 (16.0%) had hypertension, 94 (11.9%) had type 2 diabetes mellitus (T2DM), and 17 (2.2%) had nonalcoholic fatty liver disease (NAFLD). Hyperlipidemia was found to be associated with the lower risk of liver-related death [*P*<0.0001, hazard ratio (HR): 0.397, 95% confidence interval (CI): 0.268–0.588] and adverse outcomes (*P*<0.0001, HR: 0.487, 95% CI:0.367–0.646), while hypertension was noted as a risk factor for liver-related death (*P*=0.001, HR: 1.788, 95% CI:1.268–2.521) and adverse outcomes (*P*=0.014, HR: 1.417, 95% CI:1.074–1.869). Moreover, age ≥ 55 years old (*P*=0.005) and cirrhosis (*P*<0.0001) had superimposition effects on hypertension as a risk factor for liver-related death, while only cirrhosis (*P*<0.0001) had an effect on hypertension as a risk factor for adverse outcomes. Additionally, anti-sp100 was associated with adverse outcomes (*P*=0.013) in PBC patients with hypertension in univariate Cox regression analysis.

**Conclusion:**

Hyperlipidemia, hypertension, and T2DM were found as main metabolic risk factors in PBC in China. Hyperlipidemia indicated a benign clinical outcome of PBC, while hypertension indicated a poor outcome of PBC. Older age and cirrhosis had superimposition effects on hypertension for liver-related poor outcomes. Anti-sp100 might be associated with adverse outcomes, especially in PBC patients with hypertension.

## 1 Introduction

Primary biliary cholangitis (PBC; formerly referred to primary biliary cirrhosis) is an uncommon, chronic, cholestatic liver disease of autoimmune origin, and it is characterized by anti-mitochondrial autoantibodies (AMAs) and female preponderance with a progressive course that may extend over many decades. In the absence of treatment, it progresses to cirrhosis, liver failure, and eventually to liver transplantation or death (1). Because of the rarity of the disease, PBC has historically been predominantly reported in white females aged 40 to 50 years old, and the overall prevalence of clinical disease in various populations has been difficult to estimate and vary between 19 and 402 cases per million (2, 3). One recent systematic review and meta-analysis has shown an overall prevalence of 204.87 cases per million in China, which is significantly higher than that of Australia (34.98 cases per million) but slightly lower than that of Japan (221.01 cases per million) (4). Ursodeoxycholic acid (UDCA) is the initial drug of choice for PBC therapy, which not only improves biochemical indices but also delays histologic progression and improves survival without transplantation (3). According to the recent analysis, for the natural history in the UDCA ear (circa 1990), the 5-year accumulative incidence of liver decompensation, hepatocellular carcinoma (HCC) and death/liver transplantation in PBC patients was 6.95% (95% CI 2.07–11.83%), 1.54% (95% CI 0.9–2.19%) and 4.02% (95% CI 2.49–5.54%), respectively (4).

As liver is responsible for the virtually elimination of all excess cholesterol through direct secretion into bile and *via* conversion to bile salts, almost all chronic cholestatic liver diseases may be complicated with hyperlipidemia (5, 6). Serum lipids can be elevated in up to 80% of patients with PBC (7). OCA is a farnesoid X receptor (FXR), which can regulate bile acid synthesis, absorption, transport, secretion and metabolism, and has a net effect of a net effect of choleresis (6). It is the drug of choice for patients with poor response or intolerance to UDCA. Dysregulation of bile acid metabolism and FXR signaling in the gut-to-liver axis contributes to metabolic diseases including obesity, diabetes, and non-alcoholic fatty liver disease (NAFLD) (8). The metabolic syndrome (MetS) is a cluster of the most dangerous heart attack risk factors: diabetes and prediabetes, abdominal obesity, high cholesterol and high blood pressure (9). It is characterized by the presence of different combinations of risk factors including raised blood pressure, dyslipidemia (raised triglycerides and lowered high-density lipoprotein cholesterol), raised fasting glucose, and central obesity (10). NAFLD is considered as a hepatic component of MetS (11). Activation of FXR signaling pathways by bile acids, regulating glucose, lipid and energy metabolism, have become attractive avenue for MetS treatment (12).

Although hyperlipidemia is a metabolic risk factor, it has been confirmed to have no association with an increased risk of cardiovascular disease in PBC. Conversely, hypertension has been identified as the most significant risk factor for cardiovascular disease in PBC patients (7, 13). To date, the prevalence of hyperlipidemia, along with other metabolic risk factors such as hypertension, type 2 diabetes mellitus (T2DM), and NAFLD, in Chinese patients with PBC has not been well described. Furthermore, there have been few studies that have specifically evaluated the prognostic values of metabolic risk factors for liver-related outcomes in patients with PBC. Therefore, the purpose of this retrospective cohort study was to determine the prevalence of the main metabolic risk factors, such as hypertension, T2DM, hyperlipidemia, and NAFLD, in PBC patients and to evaluate their association with the risk of liver-related poor outcomes.

## 2 Material and methods

### 2.1 Study design and study population

This retrospective study was conducted by reviewing the medical records of patients who were admitted to Beijing You’an Hospital (Beijing, China) between July 2008 and September 2019 with serum positive for AMA and/or AMA-M2, with/without one or more autoimmune liver diseases (AILDs)-related autoantibodies and discharge diagnosis of PBC. The inclusion criteria were as follows: (i) biochemical evidence of cholestasis with an elevation of alkaline phosphatase activity; (ii) presence of AMA and/or AMA-M2, anti-gp210 or anti-sp100, histopathological evidence of non-suppurative cholangitis, and destruction of small- or medium-sized bile ducts (if a biopsy was performed); (iii) UDCA therapy was initiated once the diagnosis was made and maintained at a dose of 13–15 mg/kg during follow-up; (iv) the follow-up data were available in September 2019. The exclusion criteria were as follows: positive results of serological test for hepatitis B, C or E virus, comorbidity of drug-induced liver injury and alcoholic liver disease. This study was carried out in accordance with the recommendations of Helsinki Declaration as revised in 2013. The protocol was approved by the ethics committee of Beijing You’an Hospital,Capital Medical University. The individual consent for this retrospective analysis was waived.

### 2.2 Data collection and definitions

Baseline demographic and clinical data, including gender, age, discharge diagnosis, medical history, signs and symptoms, physical examination, biochemical indices, and serological features were documented on initial presentation. Hyperlipidemia [serum total cholesterol (TC) ≥ 5.2 mmol/L; low-density lipoprotein cholesterol (LDL-C) ≥ 3.4 mmol/L; high-density lipoprotein cholesterol (HDL-C)<1.0 mmol/L; triglyceride (TG) ≥ 1.7 mmol/L or current use of drugs for hyperlipidemia], hypertension (office blood pressure of ≥ 140/90 mm Hg, or mean 24-hour blood pressure of ≥ 130/80 mm Hg, or current use of drugs for hypertension) (14), T2DM (fasting serum glucose concentration >7.0 mmol/L, non-fasting glucose concentration >11.1 mmol/L or current use of drugs for diabetes), NAFLD and cirrhosis data of patients were obtained through investigating the patient’s list of discharge diagnosis or medication review on the electronic medical records. The diagnosis of NAFLD was based on the presence of fatty infiltration of the liver on abdominal imaging after excluding subjects with alcohol consumption history and the absence of alternative causes (e.g., excess alcohol, medications, etc.) (15, 16).

Etiology of cirrhosis was investigated with categories of PBC. Cirrhosis at presentation was assessed by computed tomography, magnetic resonance imaging or ultrasound examinations, and cirrhosis was diagnosed in accordance with the latest diagnostic criteria of the Chinese Society of Hepatology (17). Investigation of autoimmune diseases included only certain diseases limited to PBC- autoimmune hepatitis (AIH) overlap and Sjogren’s syndrome (SjS), which were common in PBC. Past hepatitis B virus (HBV) infection was defined as positive anti-hepatitis B core antibodies (anti-HBc) and negative hepatitis B surface antigen (HBsAg) (18). Inactive HBV carriers were defined by persistent HBsAg, anti-hepatitis B e-antigen (anti-HBe), low-serum HBV DNA, and normal alanine aminotransferase (ALT) levels (19).

The disease duration was defined as the time from the diagnosis of PBC to the end of the last follow-up. The duration of follow-up was defined as the time from the first visit to the end of the last follow-up prior to analysis of the data, or the date of transplantation or date of death (20). If patients could not be contacted or no information was available on their medical conditions for more than 6 months, they were classified as lost to follow-up. The following two clinical outcomes were considered to be of major interest: death (liver-related causes) and adverse outcomes, including hepatic decompensation (variceal bleeding, hepatic encephalopathy or ascites, which ever occurred first) and death (liver-related causes) or orthotopic liver transplantation (21).

### 2.3 Statistical analysis

The statistical analysis was performed using SPSS 23.0 software (IBM, Armonk, NY, USA). Continuous variables were expressed as mean ± s.d., and categorical variables were presented as the number (or percentage) of the subjects. Continuous variables were compared using the Mann-Whitney U test, and the Chi-square test or the Fisher’s exact test were applied for categorical variables. Survival rates, adverse event-free survival rates, and prognostic value of main metabolic risk factors were estimated using the Kaplan-Meier method, and compared *via* the log-rank test. Cox proportional-hazards model was used to identify main metabolic risk factors related to survival and adverse event-free survival. Stratified Kaplan-Meier survival analysis and stratified Cox regression analysis were employed to evaluate the effects of baseline clinical features on metabolic risk factors. The optimal threshold for age of liver-related death was established by plotting receiver operating characteristic (ROC) curves. Hazard ratio (HR) and 95% confidence interval (95% CI) were used for adjusting the strength of association. A two-tailed *P* < 0.05 was considered statistically significant.

## 3 Results

### 3.1 Profile of the study cohort

**Figure 1:**
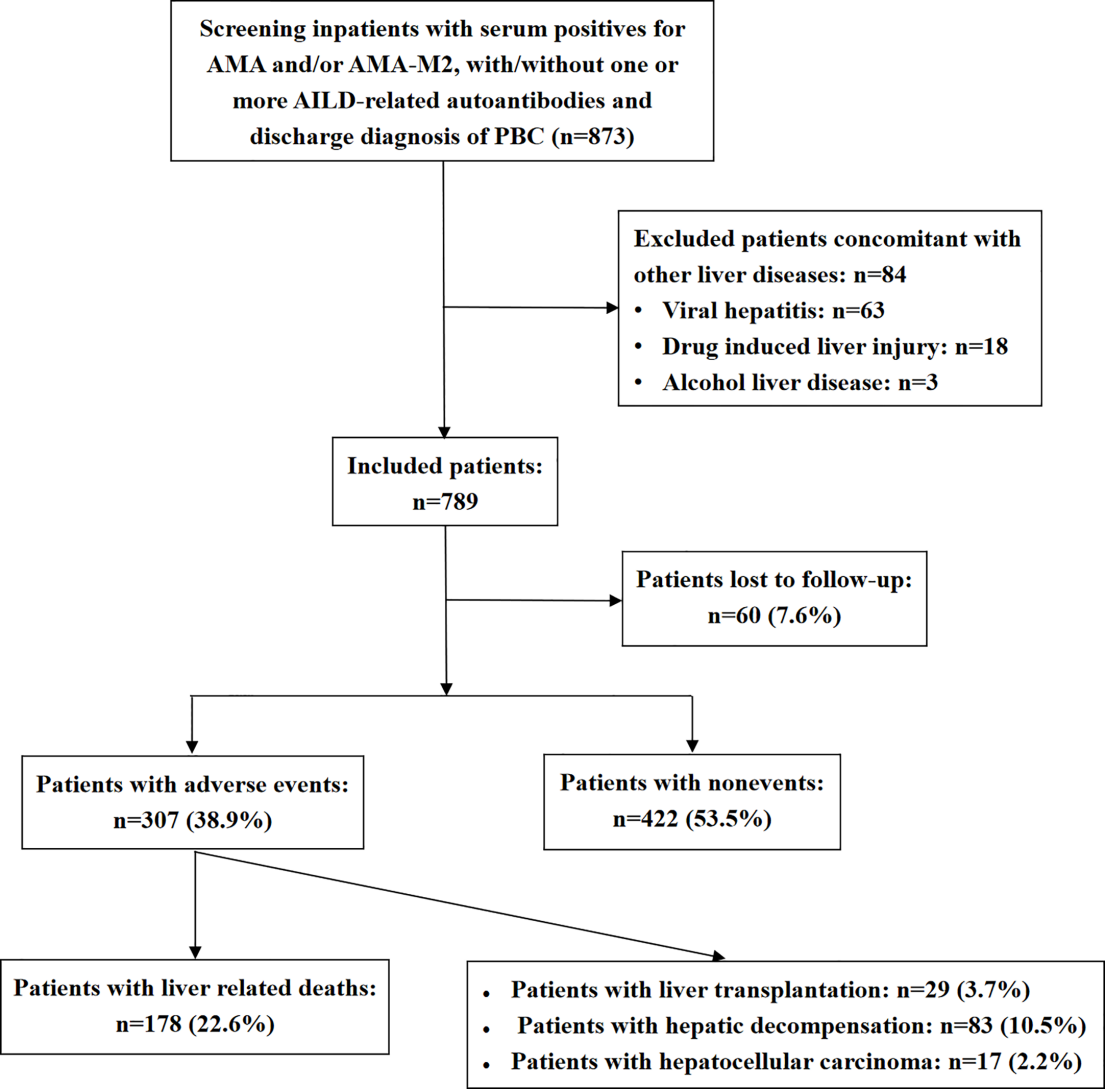
Flowchart of the patient cohort enrollment.

The study enrollment procedure is shown in **Figure 1**. Inpatients with serum positive for AMA and/or AMA-M2, with/without one or more AILDs-related autoantibodies who were admitted to Beijing You’an Hospital between July 2008 and September 2019 with discharge diagnosis of PBC were primarily enrolled in this study (n=873). Eventually, a total of 789 patients were included in the study and 84 patients were excluded.

**Figure 2:**
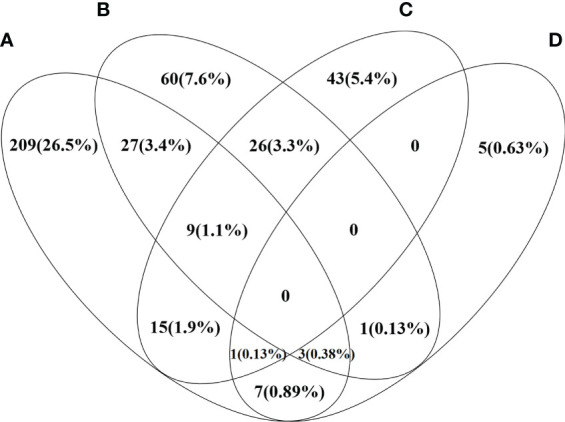
Venn diagram shows the distribution and correlation of four metabolic risk factors in the study PBC cohort. Each ring represents the number and percentage of patients with certain metabolic risk factor. **(A)** Patients with PBC and hyperlipidemia **(B)** Patients with PBC and hypertension **(C)** Patients with PBC and T2DM **(D)** Patients with PBC and NAFLD.

Patients’ baseline and follow-up data are shown in **Table 1**. Patients’ median age was 55 years old, and 689 (87.3%) patients were women. Serum AST or ALT levels were elevated [AST or ALT above the clinical laboratory upper limit of normal (ULN)] in 80.9% of patients and serum ALP >1.5×ULN, serum bilirubin > ULN, TC ≥ 5.2 mmol/L and TBA >10 µmol/L were found in 46.2%, 63.7%, 33.6%, and 78.8% of patients, respectively. Low serum ALB level [serum ALB below the clinical laboratory lower limit of normal (LLN)] and PLT<LLN were found in 73.3% and 47.0% of patients, respectively. A total of 69.0%, 53.9%, and 36.4% of patients had elevated serum IgM, IgG, and IgA levels. ANA was detected in 87.3% of patients, and 746 (94.6%) patients were found with positivity for AMA and/or AMA-M2. Of the other auto-antibodies that were common in PBC, the positive rates ranged from high to low, followed by anti-Ro52 (39.8%), anti-gp210 (36.4%), ACA and/or anti-CENP-B (23.7%), anti-sp100 (14.3%), anti-SSA (13.4%), and anti-SSB (3.4%). Notably, 454 (57.5%) patients had liver cirrhosis at baseline, 282 (41.9%) patients had history of HBV infection, and 3 (0.4%) patients were inactive HBV carriers. For the main metabolic risk factors, there were 271 (34.3%) patients with hyperlipidemia, 126 (16.0%) patients with hypertension, 94 (11.9%) patients with T2DM, and 17 (2.2%) patients with NAFLD. The coexistence and correlation of the four metabolic risk factors are shown in **Figure 2**. Other comorbidities, such as PBC-AIH overlap and SjS were relatively common in the study cohort with prevalence rates of 6.2% and 6.0%, respectively.

**Table 1:** Baseline clinical features and follow-up data of PBC patients.

Factors	All patients(n=789)	Normal ranges of laboratory tests
Age (years)	55 ± 12	
Sex		
Female, n(%)	689(87.3)	
Male, n(%)	100(12.7)	
AST or ALT>ULN, n(%)	630/779(80.9)	AST:13-35 U/L (women), 15-40 U/L (men); ALT:7-40 U/L (women), 9-50 U/L (men)
ALP>1.5×ULN, n(%)	355/768(46.2)	50-135 U/L (women), 45-125 U/L (men)
Serum bilirubin>ULN, n(%)	493/774(63.7)	5-21 µmol/L
Serum ALB<LLN, n(%)	567/774(73.3)	40-55 g/L
Total cholesterol≥5.2mmol/L	265/789(33.6)	<5.2 mmol/L
TBA>10umol/L	605/768(78.8)	<10 µmol/L
PLT<LLN	360/766(47.0)	(125-350)×10^9^/L
Immunologic features		
IgM>ULN, n(%)	458/664(69.0)	0.4-2.3 g/L
IgG>ULN, n(%)	358/664(53.9)	7.0-16.0 g/L
IgA>ULN, n(%)	242/664(36.4)	0.7-4.0 g/L
ANA, n(%)	688/788(87.3)	
AMA and/or AMA-M2, n(%)	746/789(94.6)	
ACA and/or anti-CENP-B, n(%)	187/788(23.7)	
Anti-Ro52, n(%)	236/593(39.8)	
Anti-SSA, n(%)	88/656(13.4)	
Anti-SSB, n(%)	22/656(3.4)	
Anti-gp210, n(%)	234/643(36.4)	
Anti-sp100, n(%)	92/643(14.3)	
Cirrhosis, n(%)	454/789(57.5)	
Past HBV infection, n(%)	282/673(41.9)	
Inactive HBV carriers, n(%)	3/673(0.40)	
Comorbidities		
Hyperlipidemia, n(%)	271(34.3)	
Hypertension, n(%)	126(16.0)	
T2DM, n(%)	94(11.9)	
NAFLD, n(%)	17(2.2)	
PBC-AIH overlap, n(%)	49(6.2)	
SjS, n(%)	47(6.0)	
Disease duration (months)	101 ± 59	
Duration of follow-up(months)	60 ± 32	
Lost to follow-up, n(%)	60/789(7.6)	
Clinical outcomes		
Survival, n(%)	422(53.5)	
Death, n(%)	178(22.6)	
Liver transplantation, n(%)	29(3.7)	
Hepatic decompensation, n(%)	83(10.5)	
Hepatocellular carcinoma, n(%)	17(2.2)	

ACA, anti-centromere antibody; AIH, autoimmune hepatitis; ALB, albumin; ALP, alkaline phosphatase; ALT, alanine transaminase; AMA, anti-mitochondrial autoantibody; ANA, antinuclear antibody; Anti-CENP B, anti-centromere protein B; AST, aspartate aminotransferase; GGT, gamma-glutamyl transferase; IgA, immunoglobulin A; IgG, immunoglobulin G; IgM, immunoglobulin M; LLN, lower limit of normal; NAFLD, nonalcoholic fatty liver disease; PBC, primary biliary cholangitis; PLT, platelet count; SjS, Sjogren’s syndrome; TBA, total bile acid; T2DM, type 2 diabetes mellitus; ULN, upper limit of normal.

The median duration of disease was 92 months, and the median duration of follow-up was 62 months. During the follow-up, adverse outcomes were found in 307 (38.9%) patients, including 178 (22.6%) patients who died of liver-related causes, 83 (10.5%) patients with hepatic decompensation, 29 (3.7%) patients who underwent liver transplantation, and 17 (2.2%) patients developed hepatocellular carcinoma at the end of follow-up or before death.

### 3.2 Metabolic risk factors in the PBC cohort

Comparisons of baseline clinical features and follow-up data of the study cohort stratified by with and without different metabolic risk factors are shown in **Table 2**.

**Table 2:** Comparisons of baseline clinical features and follow-up data of PBC patients stratified by metabolic risk factors.

Factors	Hyperlipidemia			Hypertension			T2DM			NAFLD		
	With (n=271)	Without (n=518)	*P*	With (n=126)	Without (n=663)	*P*	With (n=94)	Without (n=695)	*P*	With (n=17)	Without (n=772)	*P*
Age(years)	52 ± 10	59 ± 12	<0.0001	65 ± 11	55 ± 12	<0.0001	62 ± 10	56 ± 12	<0.0001	52 ± 9	57 ± 12	0.117
Sex												
Female, n(%)	236/271(87.1)	453/518(87.5)	0.883	104/126(82.5)	585/663(88.2)	0.078	81/94(86.2)	608/695(87.5)	0.720	14/17(82.4)	675/772(87.4)	0.799
Male, n(%)	35/271(12.9)	65/518(12.5)	0.883	22/126(17.5)	78/663(11.8)	0.078	13/94(13.8)	87/695(12.5)	0.720	3/17(17.6)	97/772(12.6)	0.799
AST or ALT>ULN, n(%)	239/271(88.2)	391/508(77.0)	0.0001	89/125(71.2)	541/654(82.7)	0.003	67/94(71.3)	563/685(82.2)	0.012	15/17(88.2)	615/762(80.7)	0.639
ALP>1.5×ULN, n(%)	193/271(71.2)	162/497(32.6)	<0.0001	55/124(44.4)	300/644(46.6)	0.648	38/93(40.9)	317/675(47.0)	0.268	8/16(50.0)	347/752(46.1)	0.759
Serum bilirubin>ULN, n(%)	173/271(63.8)	320/503(63.6)	0.952	78/125(62.4)	415/649(63.9)	0.742	64/94(68.1)	429/680(63.1)	0.345	8/16(50.0)	485/758(64.0)	0.250
Serum ALB<LLN, n(%)	147/271(54.2)	420/503(83.5)	<0.0001	101/125(80.8)	466/649(71.8)	0.037	81/94(86.2)	486/680(71.5)	0.003	7/16(43.8)	560/758(73.9)	0.016
Total cholesterol≥5.2mmol/L	265/271(97.8)	0/518(0)	<0.0001	38/126(30.2)	227/663(34.2)	0.374	24/94(25.5)	241/695(34.7)	0.078	11/17(64.7)	254/772(32.9)	0.006
TBA>10umol/L	212/271(78.2)	393/497(79.1)	0.784	99/124(79.8)	506/644(78.6)	0.752	78/93(83.9)	527/675(78.1)	0.200	9/16(56.3)	596/752(79.3)	0.055
PLT<LLN	61/266(22.9)	299/500(59.8)	<0.0001	58/123(47.2)	302/643(47.0)	0.970	53/92(57.6)	307/674(45.5)	0.030	2/17(11.8)	358/749(47.8)	0.003
Immunologic features												
IgM>ULN, n(%)	179/237(75.5)	279/427(65.3)	0.007	68/106(64.2)	390/558(69.9)	0.241	48/71(67.6)	410/593(69.1)	0.792	10/15(66.7)	448/649(69.0)	1.000
IgG>ULN, n(%)	106/237(44.7)	252/427(59.0)	0.0004	56/106(52.8)	302/558(54.1)	0.807	39/71(54.9)	319/593(53.8)	0.856	5/15(33.3)	353/649(54.4)	0.106
IgA>ULN, n(%)	74/237(31.2)	168/427(39.3)	0.037	44/106(41.5)	198/558(35.5)	0.237	29/71(40.8)	213/593(35.9)	0.415	3/15(20.0)	239/649(36.8)	0.181
ANA, n(%)	234/271(86.3)	454/517(87.8)	0.557	108/126(85.7)	580/662(87.6)	0.557	86/94(91.5)	602/694(86.7)	0.195	13/17(76.5)	675/771(87.5)	0.323
AMA and/or AMA-M2, n(%)	262/271(96.7)	484/518(93.4)	0.057	122/126(96.8)	624/663(94.1)	0.220	84/94(89.4)	662/695(95.3)	0.018	16/17(94.1)	730/772(94.6)	1.000
ACA and/or anti-CENP-B, n(%)	54/271(19.9)	133/517(25.7)	0.069	31/126(24.6)	156/662(23.6)	0.802	32/94(34.0)	155/694(22.3)	0.012	0/17(0)	187/771(24.3)	0.042
Anti-Ro52, n(%)	69/207(33.3)	167/386(43.3)	0.019	29/90(32.2)	207/503(41.2)	0.111	30/72(41.7)	206/521(39.5)	0.730	2/15(13.3)	234/578(40.5)	0.034
Anti-SSA, n(%)	27/233(11.6)	61/423(14.4)	0.308	10/103(9.7)	78/553(14.1)	0.229	7/80(8.8)	81/576(14.1)	0.191	3/16(18.8)	85/640(13.3)	0.793
Anti-SSB, n(%)	6/233(2.6)	16/423(3.8)	0.411	3/103(2.9)	19/553(3.4)	1.000	2/80(2.5)	20/576(3.5)	0.904	0/16(0)	22/640(3.4)	0.959
Anti-gp210, n(%)	86/228(37.7)	148/415(35.7)	0.604	35/95(36.8)	199/548(36.3)	0.921	27/71(38.0)	207/572(36.2)	0.761	4/14(28.6)	230/629(36.6)	0.539
Anti-sp100, n(%)	32/228(14.0)	60/415(14.5)	0.884	10/95(10.5)	82/548(15.0)	0.254	12/71(16.9)	80/572(14.0)	0.508	1/14(7.1)	91/629(14.5)	0.698
Cirrhosis, n(%)	99/271(36.5)	355/518(68.5)	<0.0001	86/126(68.3)	368/663(55.5)	0.008	64/94(68.1)	390/695(56.1)	0.028	3/17(17.6)	451/772(58.4)	0.001
Past HBV infection, n(%)	91/223(40.8)	191/450(42.4)	0.685	52/117(41.3)	230/556(34.7)	0.540	40/84(47.6)	242/589(41.1)	0.256	3/13(23.1)	279/660(42.3)	0.165
Inactive HBV carriers, n(%)	1/223(0.4)	2/450(0.4)	0.685	0/117(0)	3/556(0.5)	0.974	0/84(0)	3/589(0.5)	1.000	0/13(0)	3/660(0.5)	1.000
Comorbidities												
Hyperlipidemia, n(%)				39/126(31.0)	232/663(35.0)	0.381	25/94(26.6)	246/695(35.4)	0.092	11/17(64.7)	260/772(33.7)	0.008
Hypertension, n(%)	39/271(14.4)	87/518(16.8)	0.381				35/94(37.2)	91/695(13.1)	<0.0001	4/17(23.5)	122/772(15.8)	0.599
T2DM, n(%)	25/271(9.2)	69/518(13.3)	0.092	35/126(27.8)	59/663(8.9)	<0.0001				1/17(5.9)	93(772(12.0)	0.691
NAFLD, n(%)	11/271(4.1)	6/518(1.2)	0.008	4/126(3.2)	13/663(2.0)	0.599	1/94(1.1)	16/695(2.3)	0.691			
PBC-AIH overlap, n(%)	14/271(5.2)	35/518(6.8)	0.379	6/126(4.8)	43/663(6.5)	0.462	2/94(2.1)	47/695(6.8)	0.081	0/17(0)	49/772(6.3)	0.572
SjS, n(%)	12/271(4.4)	35/518(6.8)	0.189	9/126(7.1)	38/663(5.7)	0.540	6/94(6.4)	41/695(5.9)	0.852	0/17(0)	47/772(6.1)	0.595
Disease duration (months)	100 ± 49	101 ± 63	0.630	99 ± 83	101 ± 53	0.023	111 ± 74	99 ± 56	0.285	86 ± 35	101 ± 59	0.214
Duration of follow-up(months)	70 ± 28	55 ± 33	<0.0001	50 ± 34	62 ± 31	0.191	52 ± 34	61 ± 32	0.459	68 ± 18	60 ± 32	0.308
Lost to follow-up, n(%)	9/271(3.3)	51/518(9.8)	0.001	5/126(4.0)	55/663(8.3)	0.093	11/94(11.7)	49/695(7.1)	0.110	0/17(0)	60/772(7.8)	0.463
Clinical outcomes												
Survival, n(%)	202/271(74.5)	220/518(42.5)	<0.0001	56/126(44.4)	366/663(55.2)	0.026	32/94(34.0)	390/695(56.1)	<0.0001	15/17(88.2)	407/772(52.7)	0.004
Death, n(%)	30/271(11.1)	148/518(28.6)	<0.0001	44/126(34.9)	134/663(20.2)	0.0003	29/94(30.9)	149/695(21.4)	0.040	0/17(0)	178/772(23.1)	0.050
Liver transplantation, n(%)	8/271(3.0)	21/518(4.1)	0.435	1/126(0.8)	28/663(4.2)	0.106	6/94(6.4)	23/695(3.3)	0.232	0/17(0)	29/772(3.8)	0.871
Hepatic decompensation, n(%)	19/271(7.0)	64/518(12.4)	0.020	17/126(13.5)	66/663(10.0)	0.235	14/94(14.9)	69/695(9.9)	0.141	1/17(5.9)	82/772(10.6)	0.818
Hepatocellular carcinoma, n(%)	3/271(1.1)	14/518(2.7)	0.143	3/126(2.4)	14/663(2.1)	1.000	2/94(2.1)	15/695(2.2)	1.000	1/17(5.9)	16/772(2.1)	0.821

ACA, anti-centromere antibody; AIH, autoimmune hepatitis; ALB, albumin; ALP, alkaline phosphatase; ALT, alanine transaminase; AMA, anti-mitochondrial autoantibody; ANA, antinuclear antibody; Anti-CENP B, anti-centromere protein B; AST, aspartate aminotransferase; GGT, gamma-glutamyl transferase; IgA, immunoglobulin A; IgG, immunoglobulin G; IgM, immunoglobulin M; LLN, lower limit of normal; NAFLD, nonalcoholic fatty liver disease; PBC, primary biliary cholangitis; PLT, platelet count; SjS, Sjogren’s syndrome; TBA, total bile acid; T2DM, type 2 diabetes mellitus; ULN, upper limit of normal.

#### 3.2.1 Hyperlipidemia

Hyperlipidemia was found as the primary metabolic risk factor, with a prevalence rate of 34.3% (n=271). PBC patients with hyperlipidemia were significantly younger than those without hyperlipidemia (52 vs. 59 years old, *P*<0.0001). The proportions of patients with AST or ALT >ULN, ALP >1.5×ULN, serum IgM >ULN, and combined NAFLD in the PBC plus hyperlipidemia group were significantly higher than those in the non-hyperlipidemia group (all *P*<0.01). However, the proportions of patients with serum ALB<LLN, PLT< LLN, serum IgG>ULN, serum IgA>ULN, anti-Ro52-positive especially the proportions of patients with cirrhosis in the PBC plus hyperlipidemia group were significantly lower than those in the non-hyperlipidemia group (all *P*<0.05). The median duration of follow-up in the PBC plus hyperlipidemia group was longer than that in the control group (71 months vs. 57 months, *P*<0.0001), while the rate of lost to follow-up in this group was lower than that in the non-hyperlipidemia group (3.3% vs. 9.8%, *P*=0.001). For clinical outcomes, the survival rate at the end of follow-up in the PBC plus hyperlipidemia group was significantly higher than that in the non-hyperlipidemia group (74.5% vs. 42.5%, *P*<0.0001), while the mortality rate (11.1% vs. 28.6%, *P*<0.0001) and the hepatic decompensation rate (7.0% vs. 12.4%, *P*=0.020) were lower than those in the non-hyperlipidemia group.

#### 3.2.2 Hypertension

Hypertension was found as the secondary metabolic risk factor, with a prevalence rate of 16% (n=126). The median age in the PBC plus hypertension group was significantly higher than that in the non-hypertension group (64.5 years vs. 54 years, *P*<0.0001). For clinical features, the proportion of patients with AST or ALT>ULN and the median duration of disease in the PBC plus hypertension group were lower than those in the non-hypertension group (71.2% vs. 82.7%, *P*=0.003; 81.5 months vs. 94 months, *P*=0.023). However, the proportion of patients with serum ALB<LLN, especially the proportion of patients with cirrhosis as well as T2DM, were significantly higher than those in the non-hypertension group (all *P*<0.05). In the PBC plus hypertension group, the survival rate at the end of follow-up was lower than that in the non-hypertension group (44.4% vs. 55.2%, *P*=0.026), while the mortality rate was higher than that in the non-hypertension group (34.9% vs. 20.2%, *P*=0.0003).

#### 3.2.3 T2DM

T2DM was found as another common metabolic risk factor in this study cohort with the prevalence rate of 11.9% (n=94), and 35 (37.2%) patients were complicated with hypertension. For clinical features, the median age in the PBC plus T2DM group was significantly higher than that in the non-T2DM group (61.5 years vs. 55 years, *P*<0.0001). Meanwhile, the proportions of patients with serum ALB<LLN, PLT<LLN, ACA and/or anti-CENP-B-positive, cirrhosis, and hypertension in the PBC plus T2DM group were significantly higher than those in the non-T2DM group (all *P*<0.05). However, the proportions of patients with AST or ALT>ULN, AMA and/or AMA-M2-positive in the PBC plus T2DM group were lower than those in the non-T2DM group. Similar to the clinical outcomes in the PBC plus hypertension group, the survival rate at the end of follow-up was lower than that in the non-T2DM group (34.0% vs. 56.1%, *P*<0.0001), while the mortality rate was higher than that in the control group (30.9% vs. 21.4%, *P*=0.040).

#### 3.2.4 NAFLD

NAFLD was also found as a metabolic risk factor in PBC patients with a low prevalence of 2.2% (n=17) in the study cohort. PBC patients with NAFLD had lower proportions of serum ALB<LLN, PLT<LLN, anti-Ro52-positive, and cirrhosis than those in the control group (all *P*<0.05). ACA and/or anti-CENP-B was not detected in the PBC plus NAFLD group. However, the proportion of comorbidity of hyperlipidemia in the PBC plus NAFLD group was higher than that in the control group (64.7% vs. 33.7%, *P*=0.008). The clinical outcomes of PBC patients with NAFLD were the same as those combined with hyperlipidemia, indicating a higher survival rate (88.2% vs. 52.7%, *P*=0.004) and a lower mortality rate (0% vs. 23.1%, *P*=0.050) compared with the control group.

### 3.3 Prognostic value of main metabolic risk factors in PBC

**Figure 3:**
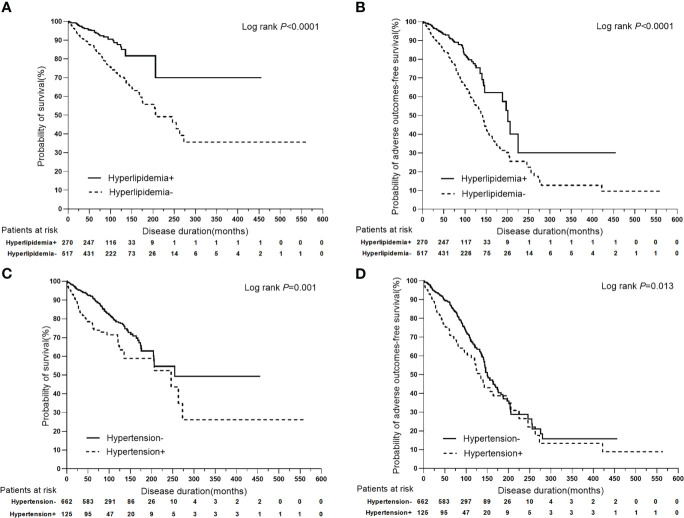
Kaplan-Meier plots for predicting clinical outcomes of PBC patients stratified by hyperlipidemia and hypertension. **(A)** Kaplan-Meier plots for predicting survival probability of PBC patients stratified by hyperlipidemia. **(B)** Kaplan-Meier plots for predicting adverse outcomes-free survival probability of PBC patients stratified by hyperlipidemia. **(C)** Kaplan-Meier plots for predicting survival probability of PBC patients stratified by hypertension. **(D)** Kaplan-Meier plots for predicting adverse outcomes-free survival probability of PBC patients stratified by hypertension.

Among the main metabolic risk factors at baseline, univariate analysis demonstrated that the presence of hyperlipidemia was associated with a lower risk of liver-related death (*P*<0.0001, HR: 0.397, 95%CI: 0.268–0.589) and adverse outcomes (*P*<0.0001, HR: 0.487, 95% CI:0.367–0.646), while hypertension at presentation was associated with a higher risk of liver-related death (*P*=0.001, HR: 1.784, 95% CI:1.266–2.515) and adverse outcomes (*P*=0.014, HR: 1.417, 95% CI:1.075–1.869). Furthermore, the Cox proportional-hazards model identified hyperlipidemia as an independent predictor of a lower risk of liver-related death (*P*<0.0001, HR: 0.397, 95% CI:0.268–0.588) and adverse outcomes (*P*<0.0001, HR: 0.487, 95% CI:0.367–0.646); however, hypertension was associated with a higher risk of liver-related death (*P*=0.001, HR: 1.788, 95% CI:1.268–2.521) and adverse outcomes (*P*=0.014, HR: 1.417, 95% CI:1.074–1.869). The other two metabolic risk factors, T2DM and NAFLD, were not found as significant prognostic factors for clinical outcomes (**Table 3**; **Figure 3**).

**Table 3:** Univariate and multivariate Cox regression analysis of prognostic metabolic risk factors in PBC patients.

Factors	Univariate analysis			Multivariate analysis		
	HR	95%CI	*P*	HR	95%CI	*P*
Liver-related death						
Hyperlipidemia	0.397	0.268-0.589	<0.0001	0.397	0.268-0.588	<0.0001
Hypertension	1.784	1.266-2.515	0.001	1.788	1.268-2.521	0.001
T2DM	1.251	0.838-1.867	0.273			
NAFLD	0.048	0.000-6.794	0.230			
Adverse outcomes						
Hyperlipidemia	0.487	0.367-0.646	<0.0001	0.487	0.367-0.646	<0.0001
Hypertension	1.417	1.075-1.869	0.014	1.417	1.074-1.869	0.014
T2DM	1.195	0.883-1.618	0.250			
NAFLD	0.395	0.098-1.589	0.191			

CI, confidence interval; HR, hazard ratio; NAFLD, nonalcoholic fatty liver disease; T2DM, type 2 diabetes mellitus.

### 3.4 The superimposition effects of baseline risk factors on hypertension

**Figure 4:**
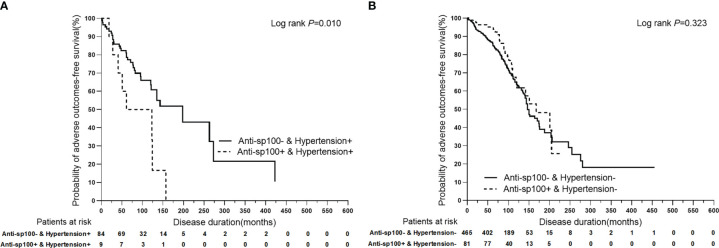
Kaplan-Meier plots for predicting adverse outcomes-free survival probability of PBC patients with/without hypertension stratified by anti-sp100. **(A)** Kaplan-Meier plots for predicting adverse outcomes-free survival probability of PBC patients with hypertension stratified by anti-sp100. **(B)** Kaplan-Meier plots for predicting adverse outcomes-free survival probability of PBC patients without hypertension stratified by anti-sp100.

To further analyze the superimposition effects of baseline risk factors on hypertension, univariate Cox regression analysis was performed on PBC patients with/without hypertension stratified by baseline clinical features (**Table 4**). Based on the findings of the univariate Cox regression analysis, there were no baseline risk factors that showed a significant association with liver-related death in the PBC plus hypertension group. However, it was observed that anti-sp100 was a distinctive risk factor that had a statistically significant association with adverse outcomes, but only in the PBC plus hypertension group (**Figure 4**) (*P*=0.013, HR: 2.686, 95% CI:1.230–5.868).

**Table 4:** Univariate Cox regression analysis of PBC patients with/without hypertension stratified by clinical features.

Factors	Hypertension	Liver-related death			Adverse outcomes		
		HR	95%CI	*P*	HR	95%CI	*P*
Sex	with/without	1.048/0.995	(0.487-2.258)/(0.597-1.657)	0.904/0.983	0.985/0.758	(0.513-1.888)/(0.500-1.148)	0.963/0.191
Age ≥ 55y	with/without	9.390/2.580	(1.289-68.382)/(1.774-3.751)	0.027/<0.0001	1.759/1.701	(0.797-3.884)/(1.308-2.213)	0.162/<0.0001
AST or ALT>ULN	with/without	1.126/1.213	(0.567-2.238)/(0.773-1.902)	0.734/0.401	1.129/1.175	(0.645-1.975)/(0.847-1.630)	0.671/0.335
ALP>1.5×ULN	with/without	0.831/0.911	(0.438-1.576)/(0.642-1.293)	0.571/0.603	1.062/0.871	(0.634-1.780)/(0.670-1.133)	0.818/0.304
Serum bilirubin>ULN	with/without	3.497/1.876	(1.611-7.593)/(1.254-2.807)	0.002/0.002	2.293/1.529	(1.310-4.016)/(1.144-2.042)	0.004/0.004
Serum ALB<LLN	with/without	5.720/12.826	(1.383-23.665)/(4.739-34.712)	0.016/<0.0001	3.383/4.416	(1.356-8.440)/(2.760-7.066)	0.009/<0.0001
TBA>10μmol/L	with/without	1.059/1.818	(0.503-2.228)/(1.091-3.029)	0.881/0.022	1.535/1.500	(0.777-3.030)/(1.051-2.141)	0.217/0.026
PLT<LLN	with/without	1.773/2.365	(0.947-3.321)/(1.636-3.418)	0.073/<0.0001	1.852/2.208	(1.101-3.115)/(1.676-2.910)	0.020/<0.0001
IgM>ULN	with/without	0.768/0.800	(0.395-1.490)/(0.534-1.197)	0.434/0.278	0.957/0.850	(0.542-1.690)/(0.627-1.152)	0.880/0.294
IgG>ULN	with/without	1.725/1.512	(0.880-3.379)/(1.018-2.247)	0.112/0.041	1.655/1.338	(0.947-2.891)/(1.000-1.790)	0.077/0.050
IgA>ULN	with/without	2.643/1.739	(1.367-5.110)/(1.184-2.552)	0.004/0.005	2.258/1.637	(1.306-3.903)/(1.228-2.183)	0.004/0.001
ANA	with/without	0.588/1.596	(0.259-1.333)/(0.881-2.893)	0.204/0.123	0.738/1.305	(0.349-1.561)/(0.873-1.951)	0.427/0.194
AMA and/or AMA-M2	with/without	1.482/0.529	(0.203-10.816)/(0.304-0.920)	0.698/0.024	2.235/0.657	(0.309-16.174)/(0.420-1.029)	0.426/0.066
ACA and/or anti-CENP-B	with/without	0.484/1.349	(0.220-1.063)/(0.934-1.948)	0.071/0.111	0.612/1.357	(0.338-1.106)/(1.032-1.786)	0.104/0.029
Anti-Ro52	with/without	0.731/0.975	(0.335-1.591)/(0.665-1.430)	0.429/0.897	0.734/1.151	(0.373-1.442)/(0.860-1.542)	0.369/0.344
Anti-SSA	with/without	1.356/1.064	(0.475-3.870)/(0.636-1.782)	0.570/0.813	0.960/1.266	(0.342-2.694)/(0.870-1.842)	0.939/0.217
Anti-SSB	with/without	1.268/1.867	(0.172-9.334)/(0.819-4.257)	0.816/0.137	0.999/1.515	(0.137-7.301)/(0.745-3.081)	0.999/0.251
Anti-gp210	with/without	1.540/1.694	(0.777-3.051)/(1.169-2.456)	0.216/0.005	1.174/1.563	(0.639-2.156)/(1.179-2.071)	0.606/0.002
Anti-sp100	with/without	2.331/0.688	(0.953-5.700)/(0.386-1.228)	0.064/0.206	2.686/0.808	(1.230-5.868)/(0.534-1.222)	**0.013**/0.312
Cirrhosis	with/without	12.607/10.070	(3.046-52.180)/(5.283-19.197)	0.0005/<0.0001	10.098/4.611	(3.663-27.838)/(3.218-6.606)	<0.0001/<0.0001
Hyperlipidemia	with/without	0.319/0.425	(0.134-0.759)/(0.273-0.661)	0.010/0.0002	0.464/0.494	(0.247-0.875)/(0.360-0.678)	0.018/<0.0001
T2DM	with/without	0.806/1.349	(0.406-1.601)/(0.819-2.221)	0.537/0.239	0.868/1.280	(0.510-1.503)/(0.885-1.852)	0.612/0.190
NAFLD	with/without	0.047/0.048	(0.000-169.304)/(0.000-19.087)	0.465/0.321	0.618/0.272	(0.085-4.487)/(0.038-1.942)	0.635/0.194
PBC-AIH overlap	with/without	0.914/1.108	(0.220-3.792)/(0.563-2.182)	0.902/0.766	1.243/1.061	(0.450-3.436)/(0.629-1.791)	0.674/0.824
SjS	with/without	1.831/1.527	(0.766-4.378)/(0.843-2.764)	0.174/0.162	1.369/1.321	(0.617-3.038)/(0.827-2.112)	0.439/0.244

Bold text denotes anti-sp100 was noted as a risk factor associated with adverse outcomes that was statistically significant only in the PBC plus hypertension group(P=0.013). ACA, anti-centromere antibody; AIH, autoimmune hepatitis; ALB, albumin; ALP, alkaline phosphatase; ALT, alanine transaminase; AMA, anti-mitochondrial autoantibody; ANA, antinuclear antibody; Anti-CENP B, anti-centromere protein B; AST, aspartate aminotransferase; CI, confidence interval; HR, hazard ratio; GGT, gamma-glutamyl transferase; IgA, immunoglobulin A; IgG, immunoglobulin G; IgM, immunoglobulin M; LLN, lower limit of normal; NAFLD, nonalcoholic fatty liver disease; PBC, primary biliary cholangitis; PLT, platelet count; SjS, Sjogren’s syndrome; TBA, total bile acid; T2DM, type 2 diabetes mellitus; ULN, upper limit of normal.

Furthermore, it was attempted to perform univariate Cox regression analysis of baseline clinical features in PBC patients stratified by hypertension (**Table 5**). It was found that PBC patients with hypertension combined with female (*P*=0.005), age ≥ 55 years (*P*=0.041), AST or ALT>ULN (*P*=0.007), ALP ≤ 1.5×ULN (*P*=0.020), serum bilirubin>ULN (*P=*0.0001), serum ALB<LLN (*P*=0.012), IgA>ULN (*P*=0.0003), cirrhosis (*P*=0.002), AMA and/or AMA-M2-postive (*P*=0.0003), anti-sp100-postive (*P*=0.0004), ACA and/or anti-CENP-B-negative (*P*<0.0001), anti-Ro52-negative (*P*=0.010), anti-SSA-negative (*P*=0.015), anti-SSB-negative (*P*=0.008), non-hyperlipidemia (*P*=0.001), non-T2DM (*P*=0.001), non-NAFLD (*P*=0.001), non-PBC-AIH overlap (*P*=0.001), and non-SjS (*P*=0.003) had a significantly higher risk of liver-related death than PBC patients with the corresponding clinical features, whereas without hypertension.

**Table 5:** Univariate Cox regression analysis of baseline clinical features in PBC patients stratified by hypertension.

Factors	Stratification variables	Liver-related death			Adverse outcomes		
		HR	95%CI	*P*	HR	95%CI	*P*
Sex	Female/male	1.722/2.004	(1.18-2.514)/(0.863-4.654)	**0.005/0.106**	1.332/1.915	(0.984-1.803)/(0.940-3.902)	0.063/0.074
Age ≥ 55y	Yes/no	1.458/0.412	(1.015-2.096)/(0.057-2.999)	**0.041/0.381**	1.202/1.256	(0.886-1.631)/(0.582-2.713)	0.238/0.561
AST or ALT>ULN	Yes/no	1.747/1.954	(1.166-2.619)/(0.978-3.908)	**0.007/0.058**	1.420/1.461	(1.023-1.971)/(0.841-2.536)	**0.036/0.178**
ALP>1.5×ULN	Yes/no	1.664/1.718	(0.953-2.904)/(1.091-2.707)	**0.073/0.020**	1.639/1.277	(1.067-2.518)/(0.879-1.856)	**0.024/0.199**
Serum bilirubin>ULN	Yes/no	2.121/1.025	(1.443-3.118)/(0.457-2.295)	**0.0001/0.953**	1.679/1.014	(1.215-2.320)/(0.577-1.784)	**0.002/0.961**
Serum ALB<LLN	Yes/no	1.569/4.038	(1.102-2.234)/(0.739-22.061)	**0.012/0.107**	1.317/1.755	(0.986-1.759)/(0.651-4.729)	0.062/0.266
TBA>10μmol/L	Yes/no	1.587/2.591	(1.075-2.344)/(1.124-5.974)	0.020/0.025	1.415/1.322	(1.043-1.921)/(0.640-2.732)	**0.026/0.451**
PLT<LLN	Yes/no	1.626/2.134	(1.053-2.511)/(1.196-3.808)	0.028/0.010	1.389/1.614	(0.977-1.973)/(1.009-2.582)	**0.067/0.046**
IgM>ULN	Yes/no	2.045/1.947	(1.265-3.306)/(1.051-3.607)	0.003/0.034	1.757/1.389	(1.203-2.566)/(0.82202.349)	**0.004/0.220**
IgG>ULN	Yes/no	2.286/1.872	(1.419-3.683)/(1.015-3.452)	0.001/0.045	1.867/1.421	(1.258-2.769)/(0.875-2.307)	**0.002/0.155**
IgA>ULN	Yes/no	2.565/1.596	(1.539-4.273)/(0.906-2.814)	**0.0003/0.106**	1.978/1.371	(1.285-3.047)/(0.883-2.127)	**0.002/0.160**
ANA	Yes/no	1.575/4.949	(1.086-2.284)/(1.861-13.162)	0.017/0.001	1.319/2.379	(0.980-1.774)/(1.061-5.335)	**0.068/0.035**
AMA and/or AMA-M2	Yes/no	1.910/0.509	(1.345-2.714)/(0.065-3.979)	**0.0003/0.520**	1.498/0.395	(1.131-1.984)/(0.052-3.011)	**0.005/0.370**
ACA and/or anti-CENP-B	Yes/no	0.699/2.347	(0.318-1.538)/(1.596-3.451)	**0.373/<0.0001**	0.658/1.827	(0.363-1.192)/(1.331-2.508)	**0.167/0.0002**
Anti-Ro52	Yes/no	1.357/1.874	(0.661-2.786)/(1.159-3.028)	**0.405/0.010**	0.875/1.571	(0.466-1.644)/(1.042-2.371)	**0.678/0.031**
Anti-SSA	Yes/no	1.513/1.642	(0.438-5.223)/(1.100-2.451)	**0.512/0.015**	0.814/1.333	(0.248-2.668)/(0.956-1.858)	0.734/0.091
Anti-SSB	Yes/no	1.001/1.681	(0.120-8.322)/(1.146-2.466)	**0.999/0.008**	0.858/1.289	(0.105-6.976)/(0.936-1.775)	0.886/0.120
Anti-gp210	Yes/no	1.590/1.724	(0.907-2.790)/(1.001-2.969)	**0.106/0.050**	1.070/1.338	(0.643-1.779)/(0.862-2.078)	0.795/0.194
Anti-sp100	Yes/no	6.189/1.434	(2.264-16.918)/(0.934-2.201)	**0.0004/0.099**	3.964/1.048	(1.762-8.921)/(0.726-1.513)	**0.001/0.804**
Cirrhosis	Yes/no	1.741/1.101	(1.225-2.475)/(0.225-5.378)	**0.002/0.905**	1.530/0.497	(1.147-2.040)/(0.171-1.439)	**0.004/0.197**
Hyperlipidemia	Yes/no	1.305/1.862	(0.528-3.225)/(1.283-2.703)	**0.565/0.001**	1.300/1.430	(0.686-2.463)/(1.050-1.946)	**0.421/0.023**
T2DM	Yes/no	1.112/1.983	(0.523-2.363)/(1.343-2.929)	**0.783/0.001**	0.901/1.596	(0.499-1.625)/(1.160-2.194)	**0.728/0.004**
NAFLD	Yes/no	(-)/1.802	(-)/(1.278-2.541)	**>0.999/0.001**	105.972/1.411	(0.000-1.910×10^9^)/(1.068-1.865)	**0.584/0.015**
PBC-AIH overlap	Yes/no	1.456/1.804	(0.314-6.753)/(1.268-2.566)	**0.631/0.001**	1.507/1.405	(0.485-4.684)/(1.056-1.869)	**0.478/0.020**
SjS	Yes/no	2.131/1.736	(0.794-5.720)/(1.204-2.504)	**0.133/0.003**	1.433/1.417	(0.569-3.610)/(1.059-1.895)	**0.446/0.019**

Bold text denotes binary categorical variables with only one P value<0.05 which were expected to be significantly associated with terminal events and then selected for multivariate analysis. ACA, anti-centromere antibody; AIH, autoimmune hepatitis; ALB, albumin; ALP, alkaline phosphatase; ALT, alanine transaminase; AMA, anti-mitochondrial autoantibody; ANA, antinuclear antibody; Anti-CENP B, anti-centromere protein B; AST, aspartate aminotransferase; CI, confidence interval; HR, hazard ratio; GGT, gamma-glutamyl transferase; IgA, immunoglobulin A; IgG, immunoglobulin G; IgM, immunoglobulin M; LLN, lower limit of normal; NAFLD, nonalcoholic fatty liver disease; PBC, primary biliary cholangitis; PLT, platelet count; SjS, Sjogren’s syndrome; TBA, total bile acid; T2DM, type 2 diabetes mellitus; ULN, upper limit of normal.

Similarly, univariate analysis indicated that PBC patients with hypertension combined with AST or ALT>ULN (*P*=0.036), ALP>1.5×ULN (*P*=0.024), serum bilirubin>ULN (*P=*0.002),TBA>10µmol/L (*P*=0.026), PLT ≥ LLN (*P*=0.046), IgM>ULN (*P*=0.004), IgG>ULN (*P*=0.002), IgA>ULN (*P*=0.002), cirrhosis (*P*=0.004), AMA and/or AMA-M2-postive (*P*=0.005), anti-sp100-postive (*P*=0.001), ANA-negative (*P*=0.035), ACA and/or anti-CENP-B-negative (*P*=0.0002), anti-Ro52-negative (*P*=0.031), non-hyperlipidemia (*P*=0.023), non-T2DM (*P*=0.004), non-NAFLD (*P*=0.015), non-PBC-AIH overlap (*P*=0.020), and non-SjS (*P*=0.019) had a significantly higher risk of adverse outcomes than PBC patients with the corresponding clinical features, while without hypertension.

**Figure 5:**
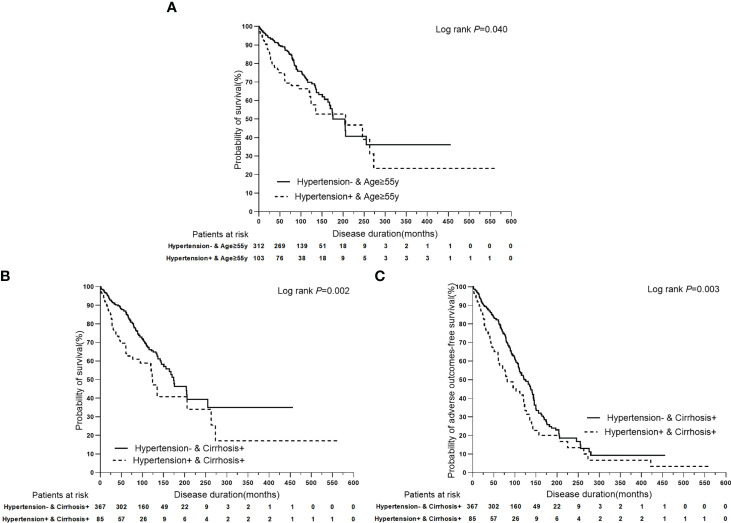
Kaplan-Meier plots for predicting clinical outcomes of PBC patients stratified by the superimposition effects of baseline clinical features on hypertension. **(A)** Kaplan-Meier plots for predicting survival probability of PBC patients with age ≥ 55y stratified by hypertension. **(B)** Kaplan-Meier plots for predicting survival probability of PBC patients with cirrhosis stratified by hypertension. **(C)** Kaplan-Meier plots for predicting adverse outcomes-free survival probability of PBC patients with cirrhosis stratified by hypertension.

In univariate Cox regression analysis of PBC with/without hypertension that stratified by clinical features, binary categorical variables with only one *P* value<0.05 were expected to be significantly associated with terminal events, and were selected for multivariate Cox regression analysis. Binary categorical variables with both *P* values<0.05 or *P* values>0.05 in univariate analysis were considered to have no effect on hypertension in patients with PBC. Multivariate Cox regression analysis indicated that age ≥ 55 years (*P*=0.005, HR: 1.893, 95% CI:1.211–2.957) and cirrhosis (*P*<0.0001, HR: 9.650, 95% CI:4.027–23.123) had superimposition effects on hypertension as risk factors for liver-related death. Similarly, only cirrhosis had a superimposition effect on hypertension as a risk factor for adverse outcomes (*P*<0.0001, HR: 6.744, 95% CI:4.218–10.781) (**Table 6**; **Figure 5**).

**Table 6:** Multivariate Cox regression analysis of the superimposition effects of baseline clinical features on hypertension as risk factors for poor outcomes in PBC patients.

Factors	Regression coefficient	SE	χ^2^	HR	95%CI	*P*
Liver-related death						
Age ≥ 55y	0.638	0.228	7.851	1.893	1.211-2.957	0.005
Cirrhosis	2.267	0.446	25.848	9.650	4.027-23.123	<0.0001
Adverse outcomes						
Cirrhosis	1.909	0.239	63.566	6.744	4.218-10.781	<0.0001

CI, confidence interval; HR, hazard ratio; NAFLD, nonalcoholic fatty liver disease; T2DM, type 2 diabetes mellitus.

## 4 Discussion

This large retrospective cohort study aimed to describe the prevalence of main metabolic risk factors in PBC patients, identify the prognostic values of these risk factors for liver-related poor outcomes, and investigate the effects of baseline clinical features on metabolic risk factors. It was revealed that hyperlipidemia, hypertension, and T2DM were the main metabolic risk factors in the PBC study cohort with the prevalence rates of 34.3%,16.0%, and 11.9%, respectively. Furthermore, hyperlipidemia was found to be independently associated with the lower risk of liver-related death and adverse outcomes. On the other hand, hypertension was a prognostic factor for liver-related poor outcomes in patients with PBC. In PBC patients with hypertension, the risk of liver-related death was increased when they are over 55 years old and have cirrhosis. However, only cirrhosis had a superimposition effect on hypertension for adverse outcomes in PBC patients.

### 4.1 Hyperlipidemia

Hyperlipidemia is a widely recognized risk factor for developing cardiovascular disease (CVD) and is prevalent among patients with PBC. In Italy, a research conducted by Longo M et al. has indicated that 76% of PBC patients had a serum cholesterol level exceeding 5.2 mmol/L in the early stages of the disease (7). It is important to note that the underlying mechanism of hyperlipidemia in PBC differs from that in other conditions. In this study, we identified hyperlipidemia as the primary metabolic risk factor in our cohort, with a prevalence of 34.3%. This is lower than the overall prevalence of dyslipidemia (40.40%) in adults in China in 2012 according to the Report on the Status of Nutrition and Chronic Diseases of Chinese residents (2015). On the other hand, our findings were consistent with a survey conducted in Beijing in 2008, which reported a dyslipidemia rate of 36.9% in the occupational population (22). As liver is responsible for the elimination of virtually excess cholesterol from the body, all chronic cholestatic liver diseases may be complicated with hyperlipidemia, in which serum cholesterol levels are typically elevated in patients with cholestatic liver diseases, reaching levels that can be as high as 5–10 times normal in some individuals (23). In this study, hyperlipidemia was primarily characterized by hypercholesterolemia, which accounted for 97.8% of cases. The main focus of concern has been whether hyperlipidemia affects the risk of cardiovascular disease in PBC patients. Studies have predominantly suggested that there is no increased risk of cardiovascular disease in patients with PBC and hyperlipidemia (7, 24). A possible risk of bias in these studies is the competing effect of liver-related morbidity and mortality (25). This has been challenged by a meta-analysis that identified a pooled risk ratio of coronary artery disease (CAD) in patients with PBC was 1.57 (95% CI, 1.21-2.06) (26). Despite this, there has been limited consideration given to the impact of hyperlipidemia on the prognosis of liver-related poor outcomes in PBC.

In this retrospective cohort study, we focused solely on liver-related causes to eliminate any potential bias from the competing effects of cardiovascular disease-related morbidity and mortality. Our findings revealed that hyperlipidemia was associated with a lower risk of liver-related death and adverse outcomes in PBC patients. These results suggest that hyperlipidemia could serve as an indicator for a benign clinical outcome in PBC. Conversely, PBC patients who did not exhibit hyperlipidemia tended to be older, had lower levels of serum ALB and PLT, and experienced higher rates of cirrhosis and mortality, suggesting a more severe and progressive form of the disease. These results may seem somewhat counter-intuitive. Because hyperlipidemia and PBC are two chronic diseases which would be expected to result in more severe and progressive disease than when only one disorder is present. One possible explanation is the progressive destruction of hepatocytes that eventually leads to the decreased cholesterol synthesis and diminished bile flow, resulting in a gradual decline in serum cholesterol levels (27). TC and HDL levels were reduced progressively with the severity of disease, suggesting that the reduced hepatic synthesis and intestinal absorption in end-stage PBC preponderated the lipid raising effect of impaired biliary secretion (7, 28). Therefore, PBC patients who do not have hyperlipidemia, particularly those in end-stage PBC, may not have a truly normal lipid metabolism despite having normal lipid levels. This could be due to reduced hepatic cholesterol synthesis, which indicates an increased risk of liver-related poor outcomes. Lipid levels serve as an indicator of hepatic lipid metabolism and may have prognostic value for liver-related outcomes in PBC, particularly in end-stage cases. However, it is important to note that this does not take into account the increased risk of cardiovascular disease. While patients with PBC-associated hyperlipidemia are not typically treated, those with traditional cardiovascular risk factors should be treated according to clinical guidelines. In cases where clinical equipoise persists, a review in a specialized hyperlipidemia clinic may be beneficial (29).

### 4.2 Hypertension

The study cohort revealed hypertension as another prevalent metabolic risk factor, with a prevalence of 16.0%. This rate aligns with the reported rate of 17.1% in the occupational population of Beijing (22). However, it is lower than the overall prevalence of 25.2% in adults in China in 2012, as reported in the 2015 Report on the Status of Nutrition and Chronic Diseases of Chinese residents. Hypertension often accompanies obesity, T2DM, and dyslipidemia, collectively known as metabolic syndrome (30). Currently, hypertension is considered the most significant risk factor for cardiovascular disease in PBC patients, increasing the likelihood of cardiovascular events (7, 13). However, it is unclear whether hypertension is associated with liver-related adverse outcomes in PBC patients. The present study revealed that PBC patients with hypertension tended to be older, had lower levels of serum ALB, and higher rates of cirrhosis, T2DM, and mortality. After excluding non-liver-related causes of death, a multivariate Cox regression analysis indicated that hypertension was the only metabolic risk factor with prognostic value for both liver-related death and adverse outcomes. The results of the stratified Cox regression analysis showed that age older than 55 years and cirrhosis had superimposition effects on hypertension for liver-related death. Additionally, only cirrhosis had a superimposition effect on hypertension for adverse outcomes in patients with PBC. It is crucial to acknowledge that hypertension becomes increasingly common and severe as individuals age, and aging is a risk factor for hypertension in both healthy individuals and those with underlying medical conditions. Furthermore, the significant activation of neurohormonal systems may lead to sodium and fluid retention in individuals with cirrhosis. As a result, these patients often experience increased blood and plasma volumes, and the severity of liver disease is closely linked to hemodynamic dysregulation (31–33).

In this study, it was observed that patients with both PBC and hypertension had significantly worse prognoses when also presenting with anti-sp100. However, anti-sp100 did not show any association with liver-related adverse outcomes in PBC patients without hypertension, according to univariate analysis. In some patients, antinuclear antibodies, particularly anti-glycoprotein 210 (anti-gp210) and/or anti-sp100, are present and may correlate with prognosis (34). Anti-sp100 positivity is associated with disease severity and poor prognosis in European populations (35–37), while the significance of anti-sp100 remains to be determined in different ethnicities. In this study, we categorized patients into two groups: those with hypertension and those without. We then further divided them into anti-sp100 positive and negative groups to investigate the impact of hypertension on anti-sp100. Our findings confirm and expand upon previous observations, as we discovered that the presence of anti-sp100 antibodies was associated with liver-related adverse outcomes in PBC patients who also had the metabolic risk factor of hypertension at baseline, as shown by univariate analysis. However, we did not observe significant associations between anti-sp100 antibodies and adverse outcomes in PBC patients without hypertension at baseline.

### 4.3 T2DM

In the present study, the prevalence of T2DM was found to be 11.9%, which is similar to the rate of 11.2% reported in mainland China from 2015 to 2017 (38). As a result, T2DM was identified as the third most common metabolic risk factor for PBC, following hyperlipidemia and hypertension. It is worth noting that diabetes in patients with cirrhosis can be either classical T2DM or hepatogenous diabetes, which is a consequence of liver disease (39). However, in this study, we did not differentiate between the two types of diabetes in PBC patients. As part of the metabolic syndrome, 54.3% of patients with both PBC and T2DM also had hyperlipidemia, hypertension, and NAFLD. Furthermore, PBC patients with T2DM tended to be older, had lower levels of serum ALB and PLT, and had higher rates of ACA and/or anti-CENP-B, cirrhosis, hypertension, and mortality. Studies have indicated that diabetes mellitus can aid in identifying nonalcoholic steatohepatitis (NASH) patients who may have severe liver fibrosis (40). Additionally, the presence of multiple metabolic disorders is associated with potentially progressive and severe liver disease (11). Herein, T2DM was not found to be an independent metabolic risk factor for liver-related poor outcomes in patients with PBC. However, it is recommended that patients with concomitant T2DM receive treatment as per normal practice. Active screening and management of diabetes could prove beneficial for PBC patients.

### 4.4 NAFLD

NAFLD is considered as the hepatic component of metabolic syndrome, which can be promoted by other metabolic risk factors, such as T2DM (39). In our study cohort, we observed that only a small percentage (2.2%) of PBC patients had NAFLD, indicating that it accounted for a relatively minor proportion of metabolic risk factors. Interestingly, we found that PBC patients with NAFLD had lower levels of serum ALB<LLN, PLT<LLN, and cirrhosis, but higher rates of hypercholesterolemia and survival. Herein, NAFLD did not emerge as an independent metabolic risk factor for poor outcomes in patients with PBC, in fact, individuals with isolated steatosis typically experience a benign clinical outcome. Furthermore, studies have shown that patients with both PBC and NAFLD do not exhibit any biochemical or non-invasive indications of more severe or progressive liver disease when compared to age- and sex-matched patients with NAFLD alone (41). The findings are in line with the conclusion of a recent study we conducted, which examined the clinical characteristics and prognosis of PBC patients using autoantibody clusters in real-world settings. Our study found that patients who tested positive only for AMA and/or AMAM2 had a higher incidence of NAFLD complications but a better overall disease prognosis than other PBC patients (42).

The results of these studies are also somewhat counter-intuitive. Given that NAFLD and PBC are both chronic liver diseases that impact different regions of the liver lobule, one would expect their coexistence to lead to more severe and progressive liver disease (41). If these results are validated, it raises the question of how the presence of NAFLD does not worsen the severity and progression of PBC. One possible explanation is that the diagnosis of NAFLD includes clinical, biochemical and radiographic tests can benefit the early detection of those potentially silent progressive uncommon liver disease, such as PBC. This could partly explain why, in this study, the proportion of cirrhosis in inpatients with PBC and NAFLD was significantly lower than that in patients without NAFLD, even though there was no difference in the disease duration of PBC between the two groups. On the other hand, it is worth considering whether UDCA, a pluripotent hepatoactive agent used to treat PBC, which is known to cause changes in the bile acid pool, protect cells, and regulate the immune system, could be beneficial for NAFLD. While early studies showed promise in treating NASH, a randomized controlled trial of NASH patients found that UDCA did not provide any histological benefits. As a result, UDCA is not recommended as a treatment for NASH (43). Furthermore, liver protection drugs such as silybin, dicyclol, polyene phosphatidylcholine, diammonium glycyrrhizate, and reduced glutathione are currently widely used in China and have demonstrated good safety. These drugs may also have potential effects on NASH and liver fibrosis (44, 45); however, further clinical verification is necessary (46). Ultimately, the impact of NAFLD on the clinical features and prognosis of PBC requires further investigation.

This study had several limitations. Firstly, it was a single-center retrospective cohort study. Secondly, patients in real-world conditions often had multiple metabolic risk factors, making it difficult to eliminate the risk of bias from confounding factors. Thirdly, the duration of metabolic risk factors was not available, which made it impossible to distinguish between classical T2DM and hepatogenous diabetes. Finally, the study only included hospitalized PBC patients due to incomplete baseline data for most outpatients. Additionally, more than half of these patients had cirrhosis and lacked early-stage PBC. Despite these limitations, the study’s relatively large single center sample sizes provide evidence of confidence in establishing the association between metabolic risk factors and PBC. However, further investigation is necessary through multicenter prospective studies to fully understand the effects of metabolic risk factors on PBC.

## 5 Conclusions

Hyperlipidemia, hypertension, and T2DM were found to be the most prevalent metabolic risk factors in Chinese patients with PBC in this study cohort. Interestingly, hyperlipidemia did not have an adverse impact on the prognosis of PBC; in fact, it was associated with a benign clinical outcome. Conversely, hypertension was found to have prognostic significance for liver-related poor outcomes in PBC patients, particularly in those over 55 years of age and with cirrhosis. Additionally, the presence of anti-sp100 antibodies might be associated with adverse outcomes in hypertensive PBC patients. Further research is necessary to fully understand the impact of metabolic risk factors on the prognosis of individuals with PBC.

## Data availability statement

The original contributions presented in the study are included in the article/supplementary material. Further inquiries can be directed to the corresponding authors.

## Ethics statement

The studies involving human participants were reviewed and approved by Beijing You’an Hospital, Capital Medical University. The ethics committee waived the requirement of written informed consent for participation.

## Author contributions

Study concept and design: D-TZ. Acquisition of data: D-TZ and W-MZ. Analysis and interpretation of data: D-TZ and YZ. Drafting of the manuscript: D-TZ. Critical revision of the manuscript for important intellectual content: H-PY, H-YL, YH, and YZ. Administrative, technical, or material support: H-PY, H-YL, YH, and YZ. Study supervision: H-PY, YZ and H-YL. All authors contributed to the article and approved the submitted version.
